# Predictive modeling of schizophrenia from genomic data: Comparison of polygenic risk score with kernel support vector machines approach

**DOI:** 10.1002/ajmg.b.32705

**Published:** 2018-12-04

**Authors:** Timothy Vivian‐Griffiths, Emily Baker, Karl M. Schmidt, Matthew Bracher‐Smith, James Walters, Andreas Artemiou, Peter Holmans, Michael C. O'Donovan, Michael J. Owen, Andrew Pocklington, Valentina Escott‐Price

**Affiliations:** ^1^ Medical Research Council Centre for Neuropsychiatric Genetics and Genomics, Division of Psychological Medicine and Clinical Neurosciences Cardiff University Cardiff United Kingdom; ^2^ School of Mathematics Cardiff University Cardiff United Kingdom

**Keywords:** polygenic risk score, schizophrenia, support vector machines

## Abstract

A major controversy in psychiatric genetics is whether nonadditive genetic interaction effects contribute to the risk of highly polygenic disorders. We applied a support vector machines (SVMs) approach, which is capable of building linear and nonlinear models using kernel methods, to classify cases from controls in a large schizophrenia case–control sample of 11,853 subjects (5,554 cases and 6,299 controls) and compared its prediction accuracy with the polygenic risk score (PRS) approach. We also investigated whether SVMs are a suitable approach to detecting nonlinear genetic effects, that is, interactions. We found that PRS provided more accurate case/control classification than either linear or nonlinear SVMs, and give a tentative explanation why PRS outperforms both multivariate regression and linear kernel SVMs. In addition, we observe that nonlinear kernel SVMs showed higher classification accuracy than linear SVMs when a large number of SNPs are entered into the model. We conclude that SVMs are a potential tool for assessing the presence of interactions, prior to searching for them explicitly.

## 1 INTRODUCTION

Schizophrenia has a complex, polygenic architecture in which a large number of genetic variants spanning a wide spectrum of population frequencies contribute to disease risk (International Schizophrenia Consortium et al., 2009; Lee et al., 2012). In recent years, specific schizophrenia‐associated risk variants have begun to emerge from large‐scale genomic studies (Sullivan, Daly, & O'Donovan, 2012).

The standard approach to genome‐wide association study (GWAS) data assumes an additive model, which, in statistical terms, is equivalent to looking for the main effects of variants contributing to disease risk. The assumption of additivity has been an extremely effective approach, but it is also pragmatic, since looking at the effects of many 100,000 s of single nucleotide polymorphisms (SNPs) would be rendered computationally expensive if all potential combinations of interactions were considered (Cordell, 2009). In addition, the excessive dimensionality of such an approach would require very severe statistical correction for multiple comparison testing (Polderman et al., 2015). Although testing for some interactions is now technically possible using graphical processing units (GPU) instead of central processing units (CPU; Hemani, Theocharidis, Wei, & Haley, 2011), extremely large sample sizes will be required to achieve sufficient power to detect small genetic interaction effect sizes, as are expected in most complex genetic traits, at the very low significance thresholds dictated by multiple testing correction.

The extent to which genetic interactions contribute to risk is unknown. One study, which explicitly modeled and tested gene expression traits for evidence of pairwise SNP interaction effects (Hemani et al., 2014), found strong evidence in favor of interactions. Moreover, additional analyses (Hemani et al., 2014) suggested that testing for interactions between SNPs with known main effects (e.g., genome‐wide significant SNPs) is unlikely to be the best strategy, as the majority of interactions involved SNPs that did not have a significant main effect on gene expression. Here, we sought to investigate whether a support vector machine learning (SVM) approach can identify the presence of interactions, without explicitly specifying interaction terms in regression models.

Previous studies have employed machine learning for similar purposes. The approaches used included neural networks (Tomita et al., 2004), random forest (Jiang, Tang, Wu, & Fu, 2009; Lunetta, Hayward, Segal, & Van Eerdewegh, 2004; Nicodemus, Malley, Strobl, & Ziegler, 2010), and SVMs (Fang & Chiu, 2012; Koo, Liew, Mohamad, & Salleh, 2013). SVMs were introduced by Vapnik and Chervonenkis (1981) and are widely used due to their flexibility in analyzing data with different distributions and their ability to deal with high‐dimensional data such as gene expression (Schölkopf, Tsuda, & Vert, 2004). Previously SVMs using SNPs as predictors were employed for the classification of populations (Bridges et al., 2011; Schrider & Kern, 2018). Our study attempts to use SNPs to distinguish schizophrenia patients from controls, where genetic differences between the groups are more subtle than between populations.

A landmark GWAS of schizophrenia, conducted by the Schizophrenia Working group of the Psychiatric Genomics Consortium (PGC; often referred to as the PGC2 study), identified 128 genome‐wide significant SNPs (of which 125 were autosomal), representing 108 independent loci (Schizophrenia Working Group of the Psychiatric Genomics Consortium, 2014). No statistically significant pairwise interactions were detected between genome‐wide significant (GWS) index SNPs. The prediction accuracy (Area under the Curve [AUC]) for the classification of schizophrenia patients and controls, obtained using polygenic risk scores (PRS) generated with (a) genome‐wide significant SNPs (*p* ≤ 5 × 10^−8^) and (b) SNPs with suggestive evidence for an association with schizophrenia (*p* ≤ .01), was AUC = 0.58 and 0.70, respectively. In the present study, we employ SVM algorithms which offer both linear and nonlinear modeling options and hence may account for pairwise and higher order SNP interactions, to explore a large schizophrenia case–control sample of 11,853 subjects (5,554 cases and 6,299 controls) for classification of SZ cases and controls. We use GWS SNPs and SNPs with suggestive evidence for association with schizophrenia.

Finding a suitable SVM model to accurately predict classification in the data involves mapping the original data points into a higher dimensional space via a kernel function. However, there is still no consensus in the field as to the optimal approaches, and there is a specific need for research to assess the advantages and limitations of machine learning algorithms when applied to genetic data (Ban, Heo, Oh, & Park, 2010). For example, a number of SVM kernel options exist; depending upon the specific choice of kernel, performance of the SVM can be further tuned by adjusting a number of parameters. We chose to investigate linear and radial basis function kernel (RBF) kernels as representatives of linear and nonlinear analyses. A number of nonlinear kernels exist and in the absence of specific knowledge that would suggest another choice, the RBF kernel makes a good default kernel to test a nonlinear model. A linear kernel has one hyper‐parameter C, reflecting whether the separation between the points is “strict” or “soft”, i.e., how strictly misclassifications are penalized; the RBF kernel has an additional parameter γ which controls the curvature of the decision boundary that separates the regions of classification.

In a direct comparison of the results of SVM analyses with those derived from a standard additive model, we first analyzed the 125 GWS autosomal SNPs from PGC2 (Schizophrenia Working Group of the Psychiatric Genomics Consortium, 2014) in our sample. We tested (a) whether the prediction accuracy of the (additive) PRS built upon GWS SNPs, was greater/smaller than that of SVM algorithm and (b) whether SVM can indicate potential interactions between GWS SNPs. We also explored a set of top ~5,000 independent SNPs most associated with schizophrenia in the PGC2 study (Schizophrenia Working Group of the Psychiatric Genomics Consortium, 2014), and tested those for the presence of potential interactions.

## 2 METHODS

### 2.1 Schizophrenia case–control data

We used the CLOZUK sample of 5,554 schizophrenia cases with treatment‐resistant schizophrenia receiving the antipsychotic clozapine, and 6,299 control samples. Individuals were genotyped on different arrays (see [Hamshere et al., 2013; Schizophrenia Working Group of the Psychiatric Genomics Consortium, 2014] for details) in two batches, called “Batch 1” (3,446 cases and 4,285 controls) and “Batch 2” (2,108 cases and 2,014 controls) hereafter. To reduce the batch effect bias in this study the data were imputed using 203,436 autosomal SNPs common to both batches. Standard quality control steps were undertaken (Hamshere et al., 2013), including INFO score threshold ≥0.9 for the SNP imputation, genotype missing rate < 2%, Minor Allele Frequency (MAF) ≥10%; Hardy–Weinberg Equilibrium (HWE) significance level *p* ≥ 10^−4^. In addition, the extended Major Histocompatibility Complex (MHC) region (chr6: 25–34 Mb) was removed due to the highly correlated nature of the SNPs in this region. The imputed data were converted into the most probable genotypes (probability >0.9), and Linkage Disequilibrium (LD) pruned, keeping the schizophrenia associated SNPs and removing those in LD (the window around associated SNPs 500 KB, *r* ^2^ value for the LD threshold 0.1). For SNP prioritization purposes, we used genome‐wide association summary statistic data, (available at https://www.med.unc.edu/pgc/results-and-downloads) of the PGC2 study (Schizophrenia Working Group of the Psychiatric Genomics Consortium, 2014), excluding the CLOZUK subset. As the SVM algorithms require complete data, missing genotypes were imputed using a multinomial distribution with the corresponding SNP genotypes frequencies.

A known problem when searching for gene–gene interactions in a GWAS is that it cannot be expected that SNPs with the largest main effects are also most likely to be involved in interactions. SNPs with no main effects are just as likely to be involved in an interaction. For example, the XOR and anti‐diagonal disease risk models specify a nonlinear interaction in the absence of independent main effects (Dong et al., 2008).

As a baseline case, we first focused on the top main effect SNPs (i.e., GWAS SNPs) in the SVM analyses, for which pairwise interactions were statistically tested (and not found) elsewhere (Schizophrenia Working Group of the Psychiatric Genomics Consortium, 2014). Thus, SVM models were initially built for the 125 GWS autosomal SNPs identified by PGC2 (Schizophrenia Working Group of the Psychiatric Genomics Consortium, 2014) that were available in our dataset. Data from the larger (in terms of sample size) Batch 1 genotyped sample were used to build and tune the SVM models' parameters. Data from Batch 2, and from both batches combined were then analyzed to explore the effect of sample size on the prediction accuracy of the SVM algorithm. We then also explored the top 4,998 independent schizophrenia associated SNPs (*p*‐value threshold <.01) and tested those for presence of potential hidden interactions. The choice of this number of SNPs (just under 5,000) was dictated by the sample size, to avoid the problem of overfitting the data when the number of features (SNPs) exceeds the number of individuals (Noble, 2006); however, we note that it is not strictly necessary to constrain the number of predictors to be less than the number of observations.

### 2.2 SVM

The data analyses were carried out in Python, using two main packages (scikit‐learn and pandas) for machine learning and data processing (McKinney, 2011; Pedregosa et al., 2011). To assess the potential contribution of interactions to the risk prediction of schizophrenia, we ran SVMs with the linear and radial basis function (RBF) kernels and compared the predictive performance of these models against each other and that of the PRS (see description of the PRS method below). We have chosen the linear kernel to compare the classification results with PRSs prediction/classification directly, and the commonly used RBF kernel function to exemplify a nonlinear classification (Chen et al., 2008). The inputs used for SVM were the reference allele counts for each polymorphism, standardized to have zero mean and variance 1. Initially, to build and test the model, we split the data into training (90%) and test (10%) subsets. The test subset had been kept separate from this CV procedure for the purpose of verifying the accuracy of the resulting predictive model. Then the hyper‐parameters were optimized in the training set using four‐fold split cross‐validation (CV) by the following Monte Carlo method. The hyper‐parameters (*C* and *γ*) for each model were selected by randomly sampling from exponential probability distributions *C* ~ Expon(*λ* = 1) for both kernels, and, additionally, *γ* ~ Expon(*λ* = 0.01) for the RBF kernel. These exponential probability distributions were used since our preliminary analysis trials suggested that lower values of the hyper‐parameters tended to result in higher classification accuracy. Each selection of hyper‐parameters was assessed by taking the mean score across the four folds of the training data. The near optimal hyper‐parameters *C* = 1 for the SVM‐Linear model and *C* = 0.5 and *γ* = 0.02 for the RBF kernel SVM model were selected and used for these 100 simulations below.

In order to compare the accuracy of linear and nonlinear modeling with the objective of identifying the presence of SNP x SNP interactions, the full datasets were used to build the SVM models. The data were randomly split into train/test subsets using 75%/25% proportions, then the SVM models were built and tested on a large number (100 times) of such splits for each of the different kernels to obtain distributions of accuracy scores for each model. The metric used for all of the performance assessment was the Area Under the receiver operating characteristic (ROC) curve (AUC) metric, also known (ROC) score (Metz, 1978).

### 2.3 Polygenic risk score

PRS is a method to summarize the trait variance captured by a set of genetic variants. We followed the approach previously described by the International Schizophrenia Consortium (International Schizophrenia Consortium et al., 2009). PRS analysis requires two independent datasets. For the first, summary data (effect size and *p*‐value) are sufficient as this dataset is used to select the SNPs, the risk score alleles and their genetic effects. The second dataset is used generate the PRS for each individual and requires individual genotypes. The PRS for each subject is calculated as a sum of risk alleles in the second dataset, weighted by the SNP effect sizes (*β*‐coefficients) derived from the first dataset. Logistic regression analysis is then used to assess whether the PRS distributions are different in cases and healthy individuals and to estimate the classification accuracy. This method is widely used in medical and population genetics (Escott‐Price et al., 2014; Escott‐Price et al., 2015; International Schizophrenia Consortium et al., 2009) to explore the genetic architecture of common disorders and assess the risk prediction utility. To compare the PRS approach with SVMs, we used exactly the same random splits (75%/25%) as for SVMs and generated individual PRSs in the 25% of the data, using summary results (*β*‐coefficients) from the 75% of the data. As above, this procedure was repeated 100 times.

Finally, the performances of the PRS and SVM models were compared using a *t* test for differences in mean AUC‐ROC scores for each approach, for different data classification models (PRS, SVMs with linear, and RBF kernels) in each data set (Batch 1 and Batch 2) separately, and in the combined dataset (Batch 1 + Batch 2).

## 3 RESULTS

@@Overall, the maximal prediction accuracy achieved by SVM was AUC = 0.60–0.66, indicating that this approach did not give a practically applicable model for prediction of schizophrenia using these data. The following results focus on the comparison of the performance of the linear and nonlinear prediction modeling.

### 3.1 GWAS significant SNPs

Initially, we built and tested models using the 125 GWS SNPs in the Batch 1 dataset only (3,446 cases and 4,285 controls).

**Figure 1:**
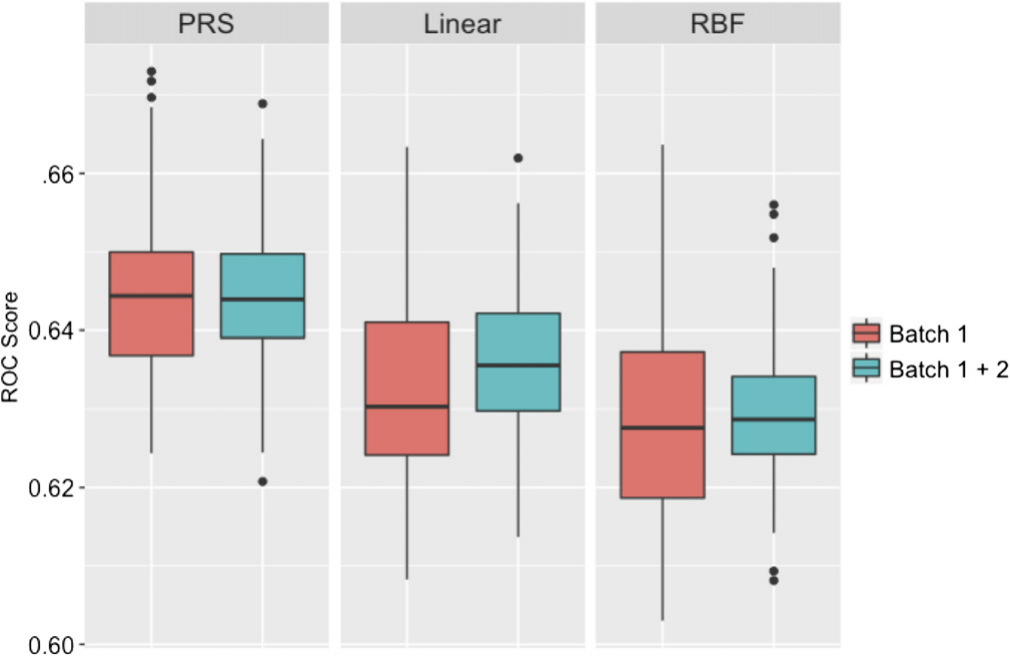
Box plots of the distribution of prediction accuracy (AUC‐ROC score, *y*‐axis) of PRS and SVM algorithms in Batch 1 data and in the combined (Batch 1 + 2 data) using 125 GWAS significant SNPs. The box plot represents the distribution of data with the horizontal line being the median, the boundaries of the box are the first and third quartiles and the extremes are minimum and maximum values on the sample [Color figure can be viewed at wileyonlinelibrary.com]

The linear kernel SVM showed similar prediction accuracy values (AUC‐ROC scores) across different values of C. Figure 1 shows the distributions of ROC scores in the Batch 1 sample for PRS and SVMs with linear (SVM‐Linear) and RBF kernels (SVM‐RBF), obtained from the best model at each cross‐validation iteration (*N* = 100).

The results (Figure 1) show that the median PRS AUC‐ROC score was 0.644, similar to previously reported results (Schizophrenia Working Group of the Psychiatric Genomics Consortium, 2014). The SVM‐Linear model performed slightly worse with a median AUC‐ROC score of 0.634, whereas the SVM‐RBF model showed the lowest accuracy, AUC‐ROC score = 0.629. The performances of the same models built on the Batch 2 data only (2,108 cases and 2,014 controls), were similar to the Batch 1 data (the median AUC‐ROC scores were 0.644, 0.630, and 0.628 for PRS, linear and RBF kernels, respectively).

To assess whether an increase in sample size improves the accuracy of the SVM models, we combined the two data sets and repeated the analyses. The results are shown in Figure 1. The results show that the accuracy of the PRS and SVM‐RBF models did not improve in the larger dataset analysis (*p* = .895 and *p* = .2, respectively), whereas in contrast, the accuracy of SVM‐Linear model did improve (*p* = .005). However, the fact that the accuracy of the RBF kernel SVM did not improve with the larger sample indicates that it is unlikely that there are detectable nonlinear effects in the data (i.e., no detectable interactions between the GWAS significant SNPs). The latter result replicates the finding of an absence of interactions between these SNPs from (Schizophrenia Working Group of the Psychiatric Genomics Consortium, 2014).

### 3.2 GWAS significant and suggestive SNPs

**Figure 2:**
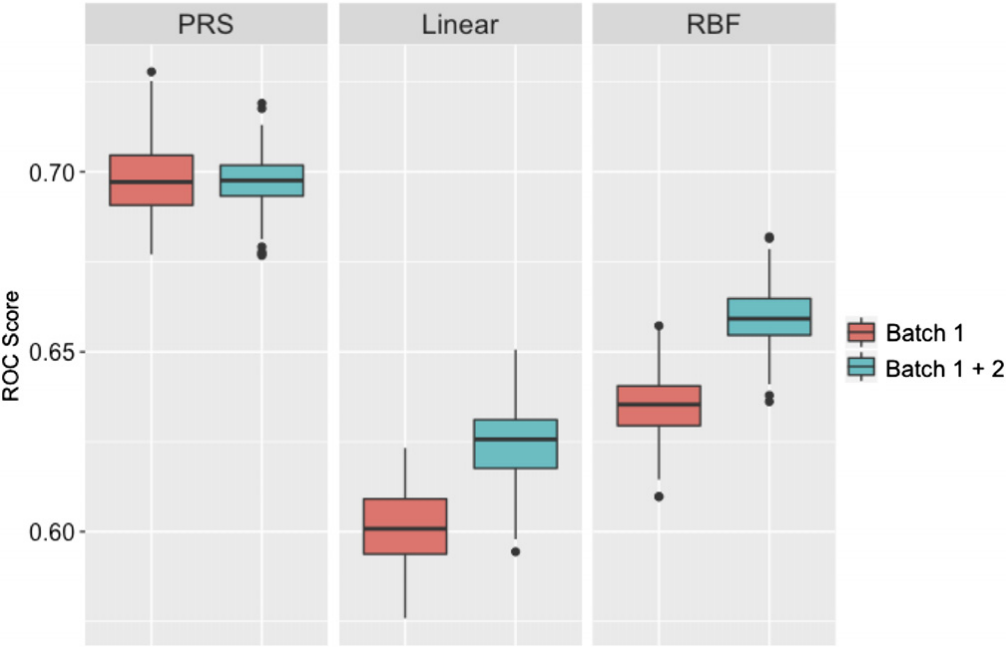
The distribution of prediction accuracy of PRS and SVM models in Batch 1 data and in the combined (Batch 1 + 2) using 4,998 SNPs [Color figure can be viewed at wileyonlinelibrary.com]

Following a similar strategy to the above, we compared the analyses on the data for 4,998 independent top SNPs, initially in the larger Batch 1 data and then in the combined dataset (Batch 1 + Batch 2). The accuracy of PRS and SVM‐RBF models improved compared to models built upon the 125 GWS SNPs (compare Figures 1 and 2). In Batch 1 data (red boxes in Figures 1 and 2), median ROC‐AUC scores increased from 0.644 and 0.629 (125 GWS SNPs) to 0.697 and 0.649 (4,998 SNPs), for PRS and SVM‐RBF models, respectively whereas the SVM‐Linear model accuracy decreased slightly (from 0.633 to 0.614). A similar pattern was observed for the Batch 2 alone (results are not shown). In the combined dataset (blue boxes in Figures 1 and 2), median ROC‐AUC scores of the SVM‐RBF model increased from 0.629 (125 GWS SNPs) to 0.662 (4,998 SNPs), and decreased from 0.635 (125 GWS SNPs) to 0.625 (4,998 SNPs) for the SVM‐Linear model. The results of the analyses of the combined dataset in comparison with just the larger of the two datasets show that the accuracy of SVM models benefited from the additional samples from the Batch 1 (*p* < 10^−16^), but the PRS model did not (*p* = .525), see Figure 1.

### 3.3 Polygenic risk scores versus multivariate regression and linear kernel SVM

**Figure 3:**
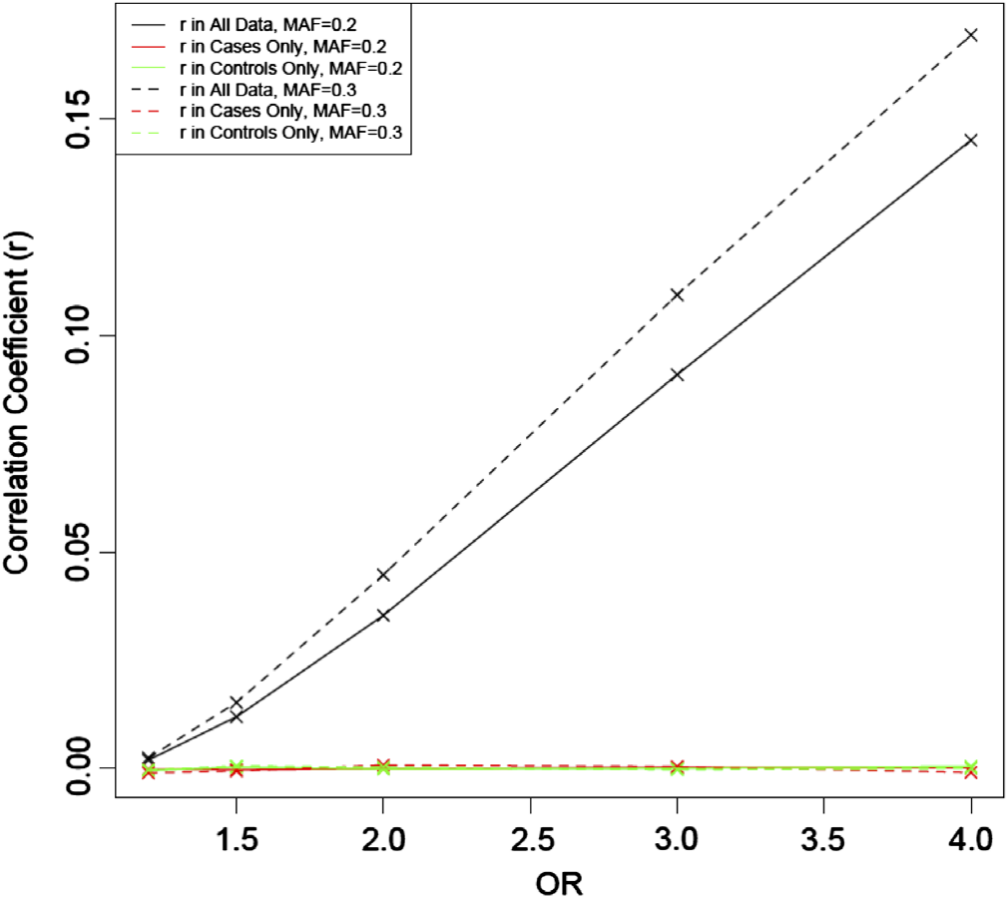
Correlation coefficient *r*, for varying ORs and MAF in case/control sample for two independent SNPs. In all data (black) and cases (red) and controls (green) separately for MAF = 0.2 (solid) and MAF = 0.3 (dashed) [Color figure can be viewed at wileyonlinelibrary.com]

**Figure 4:**
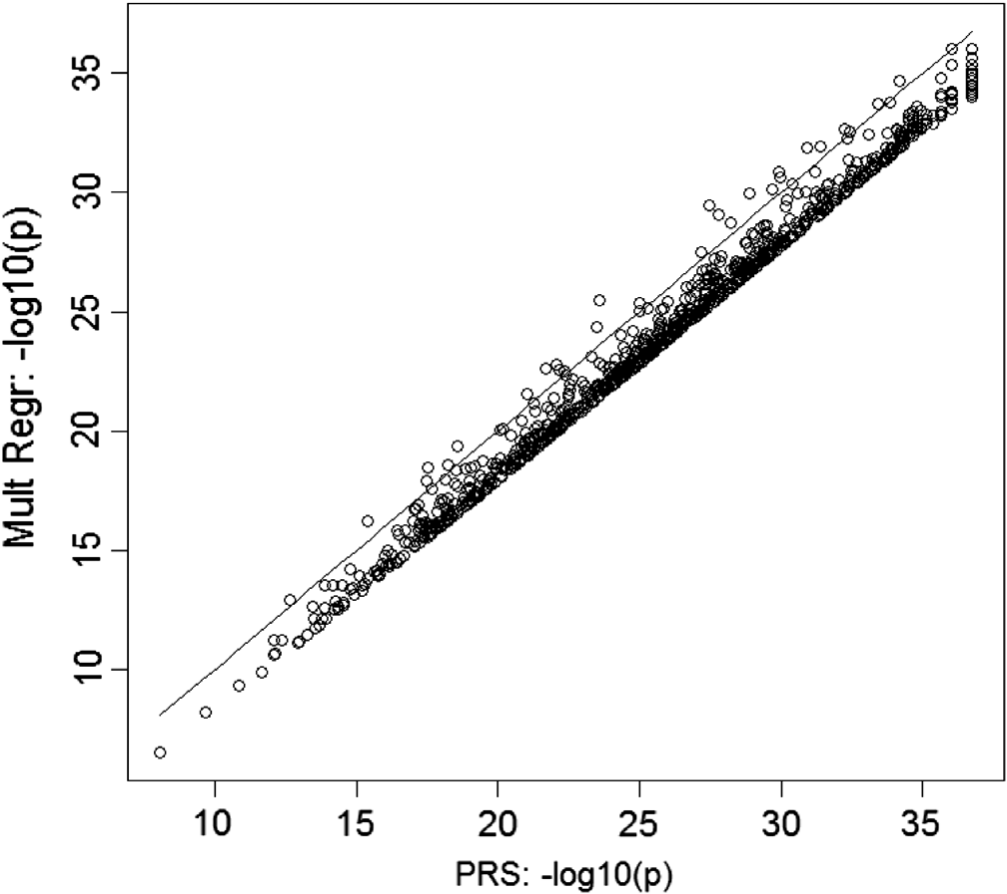
Comparison of case/control association *p*‐values when two SNPs are included as one predictor variable (PRS) in logistic regression (*x*‐axis) and separately (*y*‐axis)

We note that in the results shown, the PRS has consistently higher accuracy than the SVM models. This may be related to the fact that PRS also outperforms multivariate regression, for the following reason. Since the SNPs contributing to the PRS are prioritized for association with the disease, the risk alleles are more common among cases for each SNP. Therefore, even if associated SNPs are pruned for LD, they appear to be correlated in a case/control sample, because they are associated with disease. To illustrate this effect, we ran 1,000 simulations generating two independent SNPs which were both associated with disease in 10,000 individuals. The strength of each SNP's association to disease was varied from OR = 1 to OR = 4 and the effect of each SNP on disease risk was independent of the genotypes present at the other. Both SNPs had a MAF of either 0.2 or 0.3. The correlation in the whole sample, and in cases and controls separately was computed for each simulation. The average of these values was found across all 1,000 simulations. Figure 3 shows that separately in cases only and controls only, the correlation coefficients are approximately zero, suggesting that SNPs are independent. However, there is a larger correlation in the combined case/control data, with correlation coefficient r increasing as the strength of association and MAF increase. Figure 4 shows the direct comparison between −log_10_(*p*‐values) obtained with multivariate logistic regression (model including each SNP as an independent variable) and PRS for two independent SNPs with effect sizes OR = 1.2 and MAF = 0.3 in 1,000 simulations. The *p*‐values for PRS analyses are systematically lower than for multivariate regression. The effect is even more pronounced when the number of SNPs increases. We also compared the prediction accuracy of the linear kernel SVM and multivariate logistic regression models. The accuracy, as expected, was very similar (results are not shown). Since both the multivariate regression and SVM‐Linear models include all SNPs as separate variables for prediction, a direct comparison of the SVM‐Linear model with the multivariate regression analysis is natural. However, such comparison was not viable in our schizophrenia samples due to the limitation on the number of predictor variables in multiple linear regression modeling for accurate estimation of regression coefficients (Austin & Steyerberg, 2015), and therefore we used PRS as a proxy.

## 4 DISCUSSION

The aim of this study was to examine the application of linear and nonlinear SVMs to identifying the presence of genetic interactions which may contribute to the risk of schizophrenia, and to compare their prediction accuracy with polygenic risk score approach.

The results from genome‐wide significant SNPs indicate that since RBF (nonlinear) SVM models did not improve the prediction accuracy over the SVM‐Linear model. This implies that it is unlikely that there are SNP × SNP interactions among this set of SNPs with sufficiently large interaction effect sizes to be detected with the current sample size. When expanding analyses to a larger set of SNPs, for the Batch 1 dataset the performance of the linear kernel decreased, while that of the RBF kernel showed a modest increase. The addition of the data samples from Batch 2 resulted in a clear increase in prediction accuracy in both models. This increased benefit of including the additional samples was not seen in the models using only the GWS SNPs. In addition, we observed that the SVM model with RBF kernel displayed superior performance over the linear kernel, indicating possible evidence for interactions between the SNPs. In contrast, the prediction accuracy of PRS did not increase with the sample size. To corroborate this evidence, it would be informative to explore whether the SNPs contributing most to SVM classification tend to contain interaction effects; however, current nonlinear SVM implementations do not naturally provide information on which SNPs these are.

Neither of the SVM models demonstrated a better predictive accuracy than the polygenic risk score approach. However, we have shown that due to the selection of SNPs for the PRS, the PRS approach will have an advantage in terms of statistical power and prediction accuracy over multivariate analyses, including SVM with linear kernel and multivariate regression analyses. We note that the PRS score can be adjusted to remove the SNP–SNP correlation in the data, which makes the PRS approach essentially equivalent to multivariate regression (Yang et al., 2012). It is also possible to remove only LD between SNPs, but retain the correlation due to association by using the POLARIS method (Baker et al., 2018).

In conclusion, the RBF kernel prediction accuracy shows substantial improvements over the results from the linear kernel in real data with 4,998 SNPs. This finding indicates the potential presence of interactions between these relatively weakly associated schizophrenia genetic variants, over and above the main effects of these SNPs, whilst we found no evidence for interactions among genome‐wide significant index SNPs. Based on the results of the present study, we conclude that PRS remains the better option for the purpose of classification of schizophrenia cases from controls, although its currently demonstrated accuracy is insufficient for clinical practice.

## CONFLICT OF INTEREST

The authors have no conflict of interest to declare.
